# The Interferon Response Inhibits HIV Particle Production by Induction of TRIM22

**DOI:** 10.1371/journal.ppat.1000007

**Published:** 2008-02-29

**Authors:** Stephen D. Barr, James R. Smiley, Frederic D. Bushman

**Affiliations:** 1 Department of Medical Microbiology and Immunology, University of Alberta, Alberta Institute for Viral Immunology, Edmonton, Alberta, Canada; 2 Department of Microbiology, University of Pennsylvania School of Medicine, Philadelphia, Pennsylvania, United States of America; Feinberg School of Medicine, Northwestern University, United States of America

## Abstract

Treatment of human cells with Type 1 interferons restricts HIV replication. Here we report that the tripartite motif protein TRIM22 is a key mediator. We used transcriptional profiling to identify cellular genes that were induced by interferon treatment and identified TRIM22 as one of the most strongly up-regulated genes. We confirmed, as in previous studies, that TRIM22 over-expression inhibited HIV replication. To assess the role of TRIM22 expressed under natural inducing conditions, we compared the effects of interferon in cells depleted for TRIM22 using RNAi and found that HIV particle release was significantly increased in the knockdown, implying that TRIM22 acts as a natural antiviral effector. Further studies showed that TRIM22 inhibited budding of virus-like particles containing Gag only, indicating that Gag was the target of TRIM22. TRIM22 did not block the release of MLV or EIAV Gag particles. Inhibition was associated with diffuse cytoplasmic staining of HIV Gag rather than accumulation at the plasma membrane, suggesting TRIM22 disrupts proper trafficking. Mutational analyses of *TRIM22* showed that the catalytic amino acids Cys15 and Cys18 of the RING domain are required for TRIM22 antiviral activity. These data disclose a pathway by which Type 1 interferons obstruct HIV replication.

## Author Summary

Interferons are produced by cells in response to challenge by foreign pathogens such as viruses. The molecular mechanisms by which Type I interferons (e.g., IFNβ) inhibit the replication of HIV-1 are not fully clarified. We identified a gene called *TRIM22* that belongs to the tripartite motif (TRIM) family that was strongly induced by IFNβ. Using RNA interference to reduce the expression of TRIM22, we showed that TRIM22 is a key mediator of the IFNβ response when expressed at natural levels. We demonstrate that TRIM22 blocks the intracellular trafficking of the viral structural protein Gag to the surface of the cell, and that the antiviral activity of TRIM22 is dependent on two cysteine residues (Cys15 and Cys18) that are critical for the E3 ligase activity of RING-containing proteins. This report describes a mechanism by which Type I interferons block HIV-1 replication.

## Introduction

The interferon system is a well studied branch of the innate immune system active against viruses. Infection of vertebrate cells by many viruses provokes synthesis and secretion of interferons (IFNs), which mediate induction of a cellular antiviral state that obstructs further viral spread. Type I IFNs (α and β) are produced by many cell types, while Type II IFN (gamma) is produced by immune cells. IFN-induced signaling pathways begin with IFN binding to IFN receptors at the cell surface. This results in signal transduction via the Jak/Stat pathway, which leads to activation of interferon-responsive genes and synthesis of effector proteins, including PKR, RNAseL, Mx, and many others (reviewed in [1],[2],[3],[4]).

For HIV, previous studies have implicated IFNs in blocking both early and late stages of the HIV-1 lifecycle [5],[6],[7],[8],[9],[10]. In many cell types, the inhibition of later steps in the life cycle following integration seems to be most potent (reviewed in [2]). Although the effector mechanisms acting late have not been fully clarified, one report suggested that the IFN-inducible protein ISG15 interfered with the endosomal trafficking pathway used by HIV-1 to exit 293T cells by blocking the interaction of TSG101 with HIV-1 Gag [11].

Many tripartite motif (TRIM) proteins may function in innate immunity to restrict viral replication. TRIM5α from rhesus macaque (RhTRIM5α) blocks early replication steps of HIV-1 and other retroviruses (reviewed in [12]); in addition, one group has proposed that RhTRIM5α acts late during HIV replication as well [13]. The early target of RhTRIM5α is the capsid (CA) protein, which forms the protein shell of the viral core. Anti-HIV-1 activity has also been reported for other TRIM family members. TRIM1 has been shown to target the CA protein at an early stage pre-reverse transcription, TRIM22 has been suggested to affect HIV transcription and TRIM19 and TRIM32 have been suggested to affect trafficking of viral proteins (reviewed in [14]). Recently, TRIM28 was shown to repress transcription from a retroviral promoter by binding to proviral DNA [15].

TRIM proteins contain a highly conserved RBCC motif comprised of a RING domain, one or two B-box domains, and a predicted coiled-coil region (reviewed in [14]). The RING domain contains a specialized zinc finger [16] and has been shown to possess E3 ubiquitin ligase activity [17],[18],[19],[20],[21],[22]. Little is known about the B-box domain, which is unique to TRIM proteins. The coiled-coil domain is believed to promote protein oligomerization [23]. Several TRIM proteins also contain a C-terminal SPRY domain that is proposed to be involved in protein-protein interactions and RNA binding [24],[25].

Many TRIM family members are inducible by IFNs, providing candidate mediators of IFN inhibition. In an effort to identify TRIM proteins mediating anti-retroviral activity, we carried out transcriptional profiling of IFN-treated human osteosarcoma (HOS) cells and found that TRIM22 was the most up-regulated TRIM queried on the microarray used. HOS-CD4/CXCR4 cells were studied because they support robust HIV replication and show a strong antiviral response to IFNβ. Human TRIM22 (also known as Staf-50) was previously identified from a search for IFN-regulated genes in Daudi cells. Over-expression of TRIM22 was reported to repress transcription from the HIV-1 LTR in a plasmid reporter system in COS-1 cells [26]. In a later study by another group, over-expression of TRIM22 was reported to inhibit HIV-1 replication in human macrophages and 293T cells and postulated to act by repressing transcription from the HIV-1 LTR [27]. TRIM22 has also been implicated in normal haematopoietic differentiation [28],[29] and in diseases such as systemic lupus erythematosus [30] and Wilms tumor [31]. The expression of TRIM22 is altered in response to a variety of stimuli in addition to IFN including T-cell co-stimulation by CD2 and CD28 [32], viral antigens or infection [27],[33],[34],[35] and inflammatory cytokines [28].

Here we investigate the mechanism by which TRIM22 inhibits HIV infection. We mapped the primary block to late stages of assembly and release in the viral life cycle using over-expression studies. Short hairpin RNA against TRIM22 substantially reversed the inhibition of late steps induced by IFN treatment, implicating TRIM22 as a functional effector when expressed at biological levels. TRIM22 blocked the release of HIV-1 Gag-only particles, and the release of HIV particles was dependent on the RING domain of TRIM22. These findings clarify an IFN-inducible effector arm that disrupts the late stages of HIV infection.

## Results

### TRIM22 restricts HIV-1 replication in HOS-CD4/CXCR4 cells

Human osteosarcoma cells modified to express CD4 and CXCR4 (HOS-CD4/CXCR4) support robust HIV-1 replication, which is potently restricted by pre-treatment with IFNβ. We wished to identify restriction factors induced by IFNβ, and so carried out transcriptional profiling of HOS-CD4/CXCR4 cells before and after IFNβ treatment. As expected, a large number of genes were induced (52 total with a false discovery rate of 7%; Table S1), including the well-known antiviral genes OAS1 (∼235-fold), MX1/2 (∼24-fold), ISG20 (∼37-fold), ISGF3G (∼12-fold), IRF7 (∼13-fold) and TRIM19zeta (∼18-fold).

**Figure 1:**
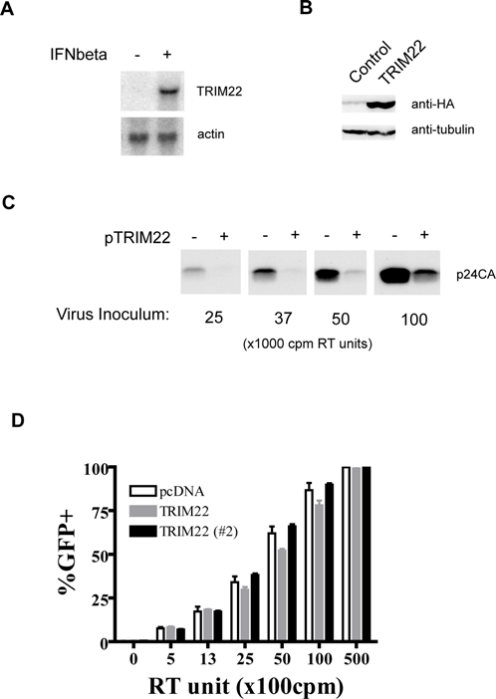
Expression of TRIM22 in HOS-CD4/CXCR4 cells blocks late steps in HIV replication. A) Induction of TRIM22 RNA levels by IFNβ treatment of HOS-CD4/CXCR4 cells. Cells were pre-treated with 1000 units/ml of IFNβ, RNA isolated and subjected to Northern blot analysis. The TRIM22 3′UTR was labeled and used as a probe. B) Stable expression of HA-tagged TRIM22 in HOS-CD4/CXCR4 cells verified by Western blotting. C) Inhibition of HIV replication in cells stably expressing TRIM22. Cells were infected with different concentrations of replication-competent HIV-1 R9. After 72 hours, virus released into the supernatant was pelleted and assayed for p24CA levels by Western blot. The numbers below the Western blots correspond to the amounts of virus added to each well, quantified according to the amount of reverse transcriptase activity. D) TRIM22 does not inhibit early HIV replication steps, subsequent to entry. The TRIM22 expressing cells were infected with different amounts of HIV-1/VSVG pseudotyped virus and analyzed by FACS for GFP expression. Data obtained from a second independently generated cell line expressing TRIM22 (#2) are also shown. Data shown are representative of two independent infections for each performed in quadruplicate (+/−StdDev).

We focused our study on *TRIM22,* which was induced at least 86-fold after IFNβ-treatment. Other *TRIM* genes that were notably up-regulated included *TRIM19* variants (2.8 to 18-fold), *TRIM21* (2.6-fold), *TRIM34* variant 1 (2.4-fold) and *TRIM5delta* (2.4-fold), consistent with previous studies [26],[36],[37],[38],[39]. Gene chip data on *TRIM22* induction was confirmed using Northern blot analysis (Figure 1A). These results support previous studies of other cell types, although we observed a higher level of IFNβ-induced *TRIM22* expression than previously reported [4],[27].

To analyze TRIM22 inhibition of HIV-1 replication, we generated HOS-CD4/CXCR4 cells that stably express hemagglutinin (HA)-tagged TRIM22 protein (Figure 1B). There was no significant difference between the doubling times of the empty vector control cells (24.4+/−2.4 hours) and the cells expressing TRIM22 (25.5+/−1.8 hours) (P = 0.7, Mann-Whitney Test), indicating TRIM22 expression was not detectably toxic. Cells stably expressing TRIM22 were then infected with increasing amounts of replication-competent HIV-1. Virus released into the supernatant after several rounds of replication was detected using p24CA antibodies in a Western blot (Figure 1C). Cells expressing TRIM22 released substantially less virus than the control cells. Similar results were obtained using another HIV-1 clone LAI (data not shown). These findings parallel a previous report [27].

### TRIM22 blocks late events of the HIV-1 lifecycle

We next analyzed the step in the HIV-life cycle affected by over-expression of TRIM22. To test for inhibition of early steps (entry through integration), we infected cells with HIV-1 pseudotyped with the VSV-G envelope and transducing *GFP*. We infected two isolates of the HOS-CD4/CXCR4 cell lines expressing TRIM22 and found no reduction in the fraction of cells positive for GFP (Figure 1D), indicating that TRIM22 did not strongly affect HIV/VSV-G early steps.

**Figure 2:**
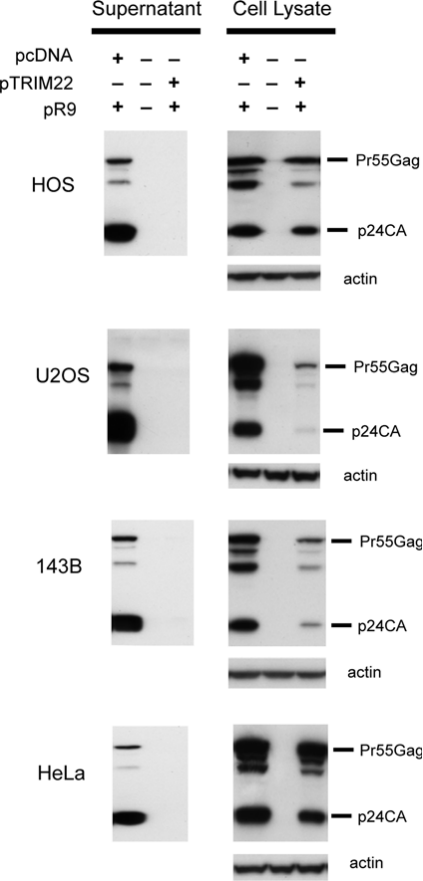
TRIM22 inhibits accumulation of viral particles in cell supernatants. HOS, U2OS, 143B and HeLa cells were studied (labeled to the left of the gels). Cells were co-transfected with pR9 (encoding full-length HIV-1) and pTRIM22 or the control empty expression vector pcDNA3.1. After 48 hours, Western blots were performed on the supernatants (left panels) and cell pellets (right panels) using p24CA antibodies.

To test whether TRIM22 interferes with late events in the HIV lifecycle, we co-transfected plasmids encoding TRIM22 and a replication-competent HIV provirus. Forty-eight hours after transfection, Western blots of cell lysates and extracellular supernatants were probed with an antibody against the p24 capsid component of the Gag polyprotein (Figure 2). Co-expression of TRIM22 blocked the release of virions from HOS-CD4/CXCR4 cells, despite the production of substantial amounts of intracellular Gag (Figure 2). Gag also failed to accumulate in the extracellular supernatant in transfected U2OS, 143B and HeLa cells.

The nature of the blocks differed among the cell types studied. After correcting with an internal transfection control (peGFP), Gag proteins accumulated to a reduced extent in U2OS and 143B cells in the presence of TRIM22, while Gag accumulated inside cells to near wild-type levels in HOS-CD4/CXCR4 and HeLa cells but were not released (Figure 2 and data not shown). In all cell types, however, accumulation of extracellular Gag was abolished by co-expression with TRIM22.

### Knocking down TRIM22 using RNA interferences boosts HIV particle production in the presence of IFNβ

**Figure 3:**
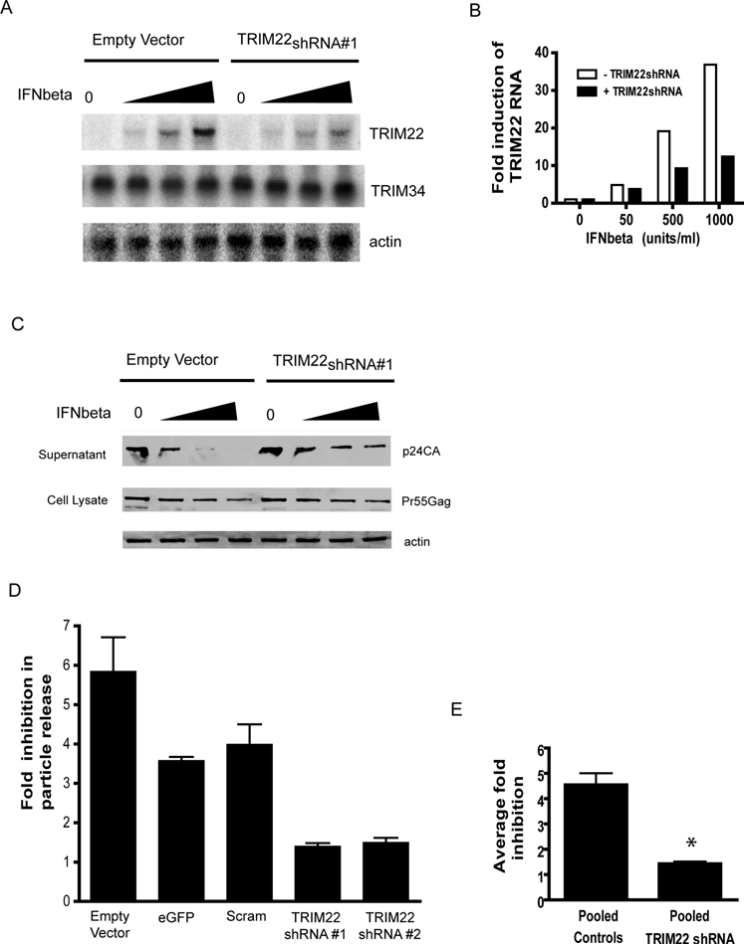
Knockdown of TRIM22 by RNA interference abrogates interferon-induced inhibition of HIV replication. A) HOS-CD4/CXCR4 cells were transfected with the empty vector control plasmid pLKO.1 or pLKO.1/TRIM22 shRNA_#1_ followed by treatment with 0, 50, 500 or 1000 units/ml of IFNβ (represented by triangles at the top of the Northern blots). Total RNA was extracted and subjected to Northern blot analysis to detect TRIM22 RNA transcripts. TRIM34 (variant 3) RNA transcript levels were also detected for a comparison (middle row). Input RNA was normalized using Northern blots probed with an actin fragment (bottom row). B) Quantitation of the Northern blots in A, indicating the fold induction of TRIM22 RNA in response to the IFNβ treatment. C) Cells were transfected with the empty vector pLKO.1 or pLKO.1/TRIM22_shRNA#1_ and treated with 1000 units/ml of IFNβ. Cells were then transfected with pR9 and Western blot analysis of HIV capsid antigen in cells and supernatants was analyzed using an anti-p24 antibody. D) Cells were transfected with the control plasmids pLKO.1, pLKO.1/eGFP_shRNA_ or pLKO.1/scrambled_shRNA_ or TRIM22 shRNA plasmids pLKO.1/TRIM22_shRNA#1_ and pLKO.1/TRIM22_shRNA#2_ and treated with 1000 units/ml of IFNβ. Following transfection with pR9, HIV capsid antigen released into the supernatant was detected by Western blot and quantified densitometrically. Data represents the averages (+/−StdDev) of at least 3 experiments performed in triplicate. E) A comparison of the average fold inhibition in particle release of the pooled control shRNAs and the pooled TRIM22 shRNAs (+/−StdDev). *, P = 0.0002 (Mann-Whitney).

The above data and results in [27] indicated that ectopic expression of TRIM22 can interfere with HIV replication, but from over-expression data alone it is uncertain whether expression at physiological levels would result in antiviral activity. For this reason, we tested whether depleting TRIM22 using short hairpin ribonucleic acid (shRNA) in the context of an interferon response allowed increased production of extracellular HIV particles. HOS-CD4/CXCR4 cells were transiently transfected for 24 hours with either pLKO.1/TRIM22_shRNA#1_ or the empty vector control (pLKO.1). In control cells, TRIM22 RNA accumulation increased in the presence of increasing concentrations of IFNβ. In the cells expressing the TRIM22 shRNA, TRIM22 RNA levels were significantly reduced (Figure 3A and B). As a control, expression of the related *TRIM34* gene was tested and found to be unaffected by the TRIM22 shRNA (Figure 3A).

The knockdown cells and the control cells were then tested for their ability to release HIV-1 virus after transfection with a plasmid encoding replication-competent HIV-1 (pR9). Accumulation of HIV Gag in cells and supernatants was monitored by Western blot using p24CA antibodies (Figure 3C). In the absence of IFNβ, the control cells released virus into the supernatant. In the presence of increasing concentrations of IFNβ, cells displayed a progressive reduction in the amount of virus released, reaching undetectable levels at 1000 units/ml of IFNβ. There was little effect of IFNβ on intracellular Gag expression levels, indicating that IFNβ acts at the level of virus assembly and/or release in HOS-CD4/CXCR4.

Cells knocked down for TRIM22, when treated with IFN, released substantially more virus into the cell supernatant than control IFN-treated cells (Figure 3C, 500 and 1000 units/ml of IFNβ). Figure 3D compares the fold decrease in particle release after treatment with 1000 units/ml of IFNβ in cells transfected with the empty vector control, an irrelevant target (eGFP) control (pLKO.1/eGFP_shRNA_), a scrambled shRNA control ( pLKO.1/scram_shRNA_) and two different TRIM22 shRNAs targeting a different region of the TRIM22 3′ UTR. Gag release was inhibited by IFN more strongly in cells transfected with each of the control shRNAs than in cells transfected with TRIM22 shRNA. Over 14 control experiments, the fold-inhibition values for 1000 units/ml IFNβ ranged from 3.0-fold to 7.4-fold. Over 8 experiments with two different TRIM22 shRNAs and 1000 u IFNβ, inhibition ranged from 1.2-fold to 1.6-fold. The difference between the means of the fold-inhibition values of TRIM22 shRNA (1.4-fold) and the pooled controls (4.5-fold) was highly significant (P = 0.0002 Mann-Whitney) (Figure 3E). These data indicate that TRIM22 is an active antiviral effector in the context of the normal response to IFNβ.

### TRIM22 inhibits budding of HIV-1 Gag-only particles

The HIV Gag polyprotein is transported to the plasma membrane after translation where it assembles into particles and buds from the cell surface. Gag protein alone is able to bud from cells when expressed in the absence of the other HIV-1 proteins (reviewed in [40]). We asked whether TRIM22 acted on the Gag polyprotein by testing inhibition of budding by Gag-only virus-like particles.

**Figure 4:**
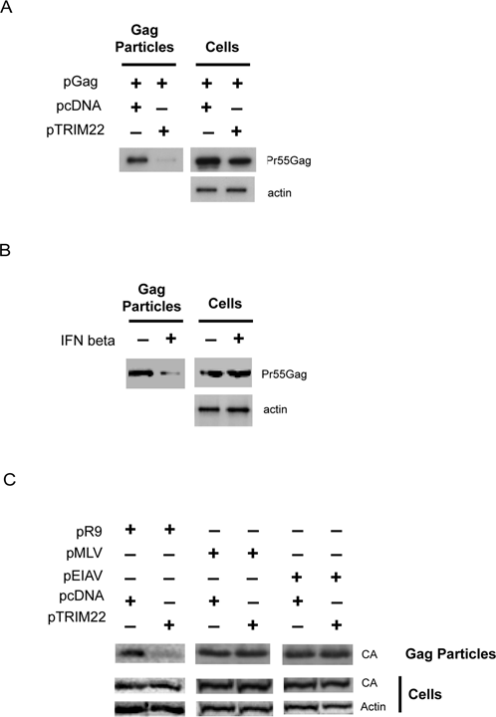
TRIM22 expression blocks release of HIV-1 Gag-only particles. A) Cells were transfected with pGag and pTRIM22 or pcDNA. Cells and Gag-only particles released into the supernatant were isolated 24 hours after transfection and analyzed by Western blotting using an anti-p24CA or anti-actin antibody. B) Cells were pre-treated for 16 hours with 1000 units/ml of IFNβ followed by transfection with pGag. Cells and Gag-only particles released into the supernatant were isolated after 24 hours and analyzed by Western blotting using an anti-p24CA antibody. C) Cells were co-transfected with pR9 or a plasmid expressing MLV GagPol (pMLV) or an EIAV packaging vector (pEIAV) with or without TRIM22 for 24 hours. Cells and Gag-containing particles were isolated and analyzed by Western blotting using an anti-capsid antibody.

We co-transfected a codon-optimized HIV Gag expression plasmid in the presence or absence of TRIM22 and measured the accumulation of Gag proteins in the cells and in culture supernatants by Western blot (Figure 4A). In the absence of TRIM22, Gag protein was detected in the cell lysate and efficiently released into the supernatant. In the presence of TRIM22, Gag was readily detected in the cell lysate, but not the supernatant. This effect parallels the effect of IFNβ on Gag-only particle release in HOS-CD4/CXCR4 cells (Figure 4B).

To assess the specificity of TRIM22, we assayed effects on murine leukemia virus (MLV) and equine infectious anemia virus (EIAV) Gag. Co-transfection of TRIM22 with either MLV Gag-Pol or EIAV Gag-Pol failed to restrict the release of Gag-containing particles into the supernatant, whereas TRIM22 restricted the release of HIV-1 Gag (Figure 4C). Together these data indicate that TRIM22 acts selectively on HIV-1 Gag.

### TRIM22 alters Gag trafficking to the plasma membrane

**Figure 5:**
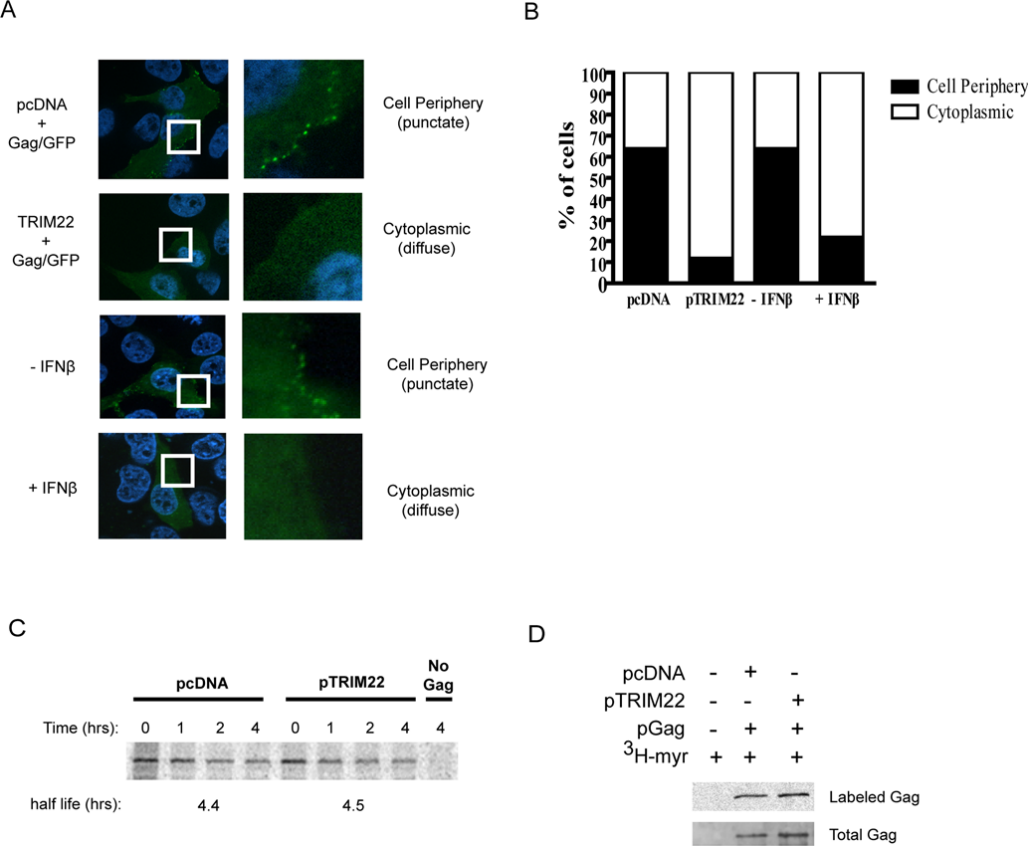
TRIM22 alters the sub-cellular localization of Gag protein. A) Analysis of Gag localization by fluorescence microscopy. HOS-CD4/CXCR4 cells were co-transfected with pTRIM22 and pGag/GFP for 3 hours, washed and incubated with 100 µM cycloheximide for 3 hours. IFNβ pre-treatment was done for 16 hours prior to transfection with pGag/GFP. Cells were washed and incubated in fresh media for a further 48 hours. Gag-GFP localization was assessed using fluorescence microscopy. High magnification images of the white squares are shown to the right. B) Quantifications of images as in A from three independent experiments. GFP-positive cells were classified as showing either punctate fluorescence at the cell periphery or diffuse cytoplasmic fluorescence. The number of cells counted was: pcDNA, n = 332; TRIM22, n = 386; −IFNβ = 497; +IFNβ = 398. C) HOS-CD4/CXCR4 cells were co-transfected with pGag in the absence or presence of pTRIM22 and pulse-chase analysis of intracellular Gag protein was done. D) Detection of ^3^H-myristic acid-labeled Gag. Cells were co-transfected with pGag in the absence or presence of pTRIM22 for 24 hours and then labeled with ^3^H-myristic acid overnight. Gag was immunoprecipitated and resolved on a SDS-PAGE gel before exposure to film (“Labeled Gag”) or Western blot analysis using anti-p24CA (“Total Gag”).

We next investigated whether TRIM22-mediated inhibition was associated with altered Gag trafficking by monitoring the subcellular localization of HIV Gag fused to GFP (pGag-GFP) [41],[42],[43],[44]. We transfected pGag-GFP in the presence or absence of pTRIM22, then 3 hours later treated the cells with cycloheximide for 3 hours. Forty-eight hours after release of the cycloheximide block, the localization of the Gag-GFP protein was visualized by fluorescence microscopy by taking optical slices through the center of cells (Figure 5A). In the control cells, 65% of GFP-positive cells had punctate fluorescence at or near the plasma membrane and 35% of the cells showed diffuse cytoplasmic fluorescence. In contrast, in cells expressing TRIM22, 12% of the cells had punctate fluorescence at or near the plasma membrane and 88% of the cells yielded diffuse cytoplasmic localization without visible puncta (Figure 5B). The observed difference in proportions was highly significant (P<0.0001, Chi-square test).

To test whether IFNβ altered Gag localization, as with TRIM22 expression alone, we compared the effects of pre-treating cells with 1000 units/ml of IFNβ followed by transfection with GagGFP and microscopy. In the absence of IFNβ, 64% of GFP-positive cells had punctate fluorescence at or near the plasma membrane and 36% of the cells showed diffuse cytoplasmic fluorescence. In contrast, 22% of the IFNβ-treated cells had punctate fluorescence at or near the plasma membrane and 78% of the cells yielded diffuse cytoplasmic localization without visible puncta (Figures 5A and 5B). The difference in the number of cells exhibiting punctate localization at the cell surface before and after IFNβ-treatment was highly significant (P<0.0001, Chi-Square) and paralleled the result with the expression of TRIM22 alone.

To determine whether the diminished accumulation of Gag at the plasma membrane is due to accelerated degradation of Gag, we performed pulse-chase analysis on intracellular Gag levels in HOS-CD4/CXCR4 cells (Figure 5C). The half life of Gag was 4.4 hours in cells transfected with the empty vector control (pcDNA) and 4.5 hours when TRIM22 was co-transfected. In addition, Western analysis also yielded similar levels of intracellular Gag protein in the presence and absence of TRIM22 (data not shown). These data indicate that inhibition of particle release by TRIM22 is associated with diminished accumulation of Gag at the plasma membrane and is not likely the result of increased turnover of Gag in HOS-CD4/CXCR4 cells.

The myristoylation of Gag is an important requirement for targeting Gag to the plasma membrane and for virus assembly and release [45],[46],[47],[48],[49]. We asked whether the TRIM22-mediated disruption of Gag trafficking is a result of a defect in Gag myristoylation. We co-expressed Gag with or without TRIM22 in the presence of ^3^H-myristic acid and immunoprecipitated Gag from the cellular extract with anti-p24CA. Gag myristoylation was readily detected both in the absence and presence of TRIM22, indicating that TRIM22 does not block the myristoylation of Gag (Figure 5D). A fraction of the immunoprecipitated protein was subjected to Western blot analysis using anti-p24CA, confirming the presence of comparable amounts of Gag protein in both of the samples.

### The E3 ligase catalytic site of TRIM22 is required for antiviral activity

The modification of proteins by E3 ligases is known to be associated with altered endocytic trafficking (reviewed in [50],[51]). Since TRIM22 alters Gag trafficking and the RING domain of TRIMs have homology with E3 ligases, we tested whether the E3 ligase active site, present in the RING domain, is required for the antiviral effects of TRIM22. We mutated two conserved cysteine residues (C15A/C18A) in the RING domain that have been shown to inactivate the ubiquitin ligase activity in other TRIMs [17],[52],[53].

**Figure 6:**
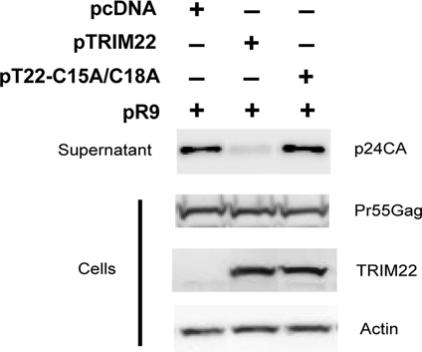
E3 ligase catalytic site is required for HIV particle release. pR9 was transfected with or without pcDNA, pTRIM22 or pT22-C15A/C18A for 24 hours. Western blots were performed on virus released into the supernatants (top panel) using anti-p24CA and on the cell pellets (lower panels) using anti-p24CA, anti-HA or anti-actin respectively.

We found that cells co-transfected with pR9 and either the empty vector control or the pTRIM22 C15A/C18A mutant did not block the release of virus, though wildtype TRIM22 blocked quite strongly (Figure 6). Gag accumulated inside cells to near wild-type levels (Figure 6, second panel) and the expression levels of wildtype TRIM22 and the C15A/C18A mutant were similar (Figure 6, second panel). These data implicate the catalytic cysteine residues at position 15 and 18 of the RING domain as important for the observed antiviral effects of TRIM22.

**Figure 7:**
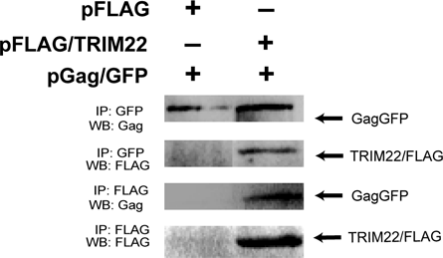
TRIM22 interacts with Gag. HOS-CD4/CXCR4 cells were co-transfected with pGag-GFP and pFLAG-TRIM22 (or empty vector control pFLAG) and immunoprecipitated with anti-GFP or anti-FLAG. Precipitated Gag and TRIM22 were detected by Western blotting using p24CA or FLAG antibodies.

We next asked whether we could detect an interaction between TRIM22 and Pr55Gag. We immunoprecipitated a Gag-GFP fusion protein expressed in the presence of FLAG-tagged TRIM22 with anti-GFP antibodies and then performed a Western blot with anti-Flag antibodies and detected co-precipitation of FLAG-TRIM22 (Figure 7). Reverse immunoprecipitation using anti-FLAG pulled down Gag-GFP only when FLAG-TRIM22 was co-expressed. Treatment of the samples with RNaseA prior to immunoprecipitation did not interfere with the association of TRIM22 with Gag, indicating that an RNA bridge did not mediate the interaction. TRIM22 did not co-immunoprecipitate with either MLV or EIAV Gag (data not shown). Thus TRIM22 binds specifically to HIV Gag.

## Discussion

Here we present data that TRIM22 is an interferon inducible effector that is responsible at least in part for the late IFNβ block to HIV-1 replication. We observed, as have others, that over-expression of TRIM22 in cells obstructed HIV replication. We show here that knockdown of TRIM22 in HOS-CD4/CXCR4 cells in the context of a natural IFN response abrogated the late block to HIV replication, implicating TRIM22 as an antiviral effector. TRIM22 expression was able to block release of HIV-1 Gag-only virus-like particles, but not MLV or EIAV Gag particles, indicating that TRIM22 acts specifically on the HIV Gag protein. In binding studies, TRIM22 associated with HIV-1 Gag but not MLV or EIAV Gag. TRIM22 expression altered Gag localization–cells containing Gag-GFP fusions showed punctate structures at the plasma membrane, potential sites of virus assembly and budding [41],[54],[55]. In contrast, Gag-GFP expressed in the presence of TRIM22 alone or IFNβ showed only diffuse staining. TRIM22 with substitutions in the E3 catalytic residues were unable to restrict HIV-1 release, indicating that inhibition was dependent on the active site. Taken together, these data implicate TRIM22 as an effector arm of the IFN-mediated antiviral state that selectively disrupts Gag sorting and release.

Complications in studying mechanisms of the IFN response arise from i) the pronounced cell type specificity of IFN action, ii) differential effects of different doses, and iii) differences among the effects of interferon types. Previous studies have reported that Type I IFNs can block HIV gene expression, Gag sorting, and Gag release [9],[11],[26],[56],[57],[58],[59],[60],[61],[62]. Here we show, using HOS-CD4/CXCR4 cells, that TRIM22 acts on HIV Gag, allowing efficient Gag expression but blocking assembly and/or release. Similar results were seen in HeLa cells. However, in U2OS and 143B cells, TRIM22 expression resulted in reduced accumulation of Gag in cells, indicating that TRIM22 inhibited either Gag synthesis or increased Gag turnover. A previous study, analyzing TRIM22 in COS cells, indicated that TRIM22 inhibited LTR-driven transcription. A similar mechanism may operate in U2OS cells and 143B cells. Recently, rhesus TRIM5α (but not its human ortholog) was shown to cause the rapid degradation of Gag in 293T cells [63]. We found no evidence that TRIM22 drastically altered the stability of Gag protein levels in HOS-CD4/CXCR4 or HeLa cells, however it is possible that altered Gag sorting results in faster degradation in U2OS and 143B cells than seen in HOS-CD4/CXCR4 and HeLa cells.

Against this background of cell type specific effects, comparisons of our results to previous reports are quite tentative. In one study, addition of 500 units/ml of IFNα to 293T cells was reported to inhibit HIV particle release about two-fold, and an siRNA against the interferon-induced ubiquitin-like molecule ISG15 blocked IFN inhibition [64],[65],[66]. In HOS-CD4/CXCR4 cells treated with IFNβ at 1000 units/ml, the inhibition of particle release was stronger (3.0-fold to 7.4-fold), but the shRNA against TRIM22 did not completely reverse IFN inhibition. One possibility is that TRIM22 and ISG15 both act in the same pathway. For example, TRIM22 might act as an E3 ligase for ISG15. Further experiments will be needed to assess the relationship of inhibition by TRIM22 to that of ISG15.

Another recent report suggested that IFNα treatment caused HIV-1 virions to become tethered to cell surfaces, resulting in eventual re-uptake by endocytosis [67]. The viral Vpu protein was reported to overcome this block in some cell types. It is possible that the mis-sorting of Gag observed here in HOS-CD4/CXCR4 cells results from virions trafficking to the membrane followed by re-uptake, though in HOS-CD4/CXCR4 cells there was no evidence for Gag accumulating at membranes at early times as might be expected from the re-uptake model. Also, in the HOS-CD4/CXCR4 cell model, expression of Gag in the presence or absence of Vpu did not noticeably affect particle accumulation in the supernatant. It will be useful to identify the cell type or experimental differences responsible for these divergent observations.

How human TRIM22 alters intracellular Gag trafficking in HOS-CD4/CXCR4 cells is unknown but a fascinating problem for further study. One candidate model would be that TRIM22 acts by interfering with Gag binding to membranes via the linked myristate residue. Membrane binding of Gag has been reported to be regulated by a multimerization-dependent myristoyl switch [68],[69],[70],[71]. We found that Gag was myristoylated normally in the presence of TRIM22 (Figure 5C) but TRIM22 might affect Gag multimerization and resulting exposure of the myristate residue. We also found that that the E3 ligase activity of TRIM22 is important for restricting viral particle release, which implies that TRIM22 probably disrupts Gag trafficking by transferring ubiquitin (or an ubiquitin-related moiety) to one or more targeted proteins. Although TRIM22 interacts with Gag, we have yet to detect a convincing effect of TRIM22 on the levels of Gag ubiquitylation or a size-altering modification of Gag (data not shown), raising the possibility that a cellular protein involved in Gag trafficking is the target. Thus studies of TRIM22 link E3 ligases, IFN, and HIV late replication steps, providing a new experimental route to investigating these issues.

## Materials and Methods

### Cell lines, plasmids and antibodies

293T, HOS-CD4/CXCR4, HeLa, 143B, and U2OS were maintained in standard growth media (Dulbecco's Modified Eagle's Medium (DMEM), supplemented with 10% heat-inactivated Fetal Bovine Serum (FBS), 100 U/ml Penicillin and 100 µg/ml Streptomycin) at 37°C with 5% CO_2_. HOS-CD4/CXCR4 cells stably expressing TRIM22 was generated by transfecting the cells with pTRIM22 followed by selection with 2 mg/ml of Genetecin. pTRIM22 was generated by cloning the TRIM22 coding region (GenBank Accession X82200) into pcDNA3.1HA using MfeI and XhoI restriction sites to generate pTRIM22. pFLAG/TRIM22 was generated by cloning the same TRIM22 coding region into p3xFLAG-CMV-10 (Sigma) using HindIII and XbaI restriction sites. pT22-C15A/C18A was generated using the QuikChange Site Directed Mutagenesis kit (Stratagene) according to manufacturer's directions. pTRIM22 was used as the template and the oligos used were 5′ gag aag gag gtg acc gcc ccc atc gcc ctg gag ctc c 3′ (forward) and 5′ gga gct cca ggg cga tgg ggg cgg tca cct cct tct c 3′ (reverse). pGag/GFP was a generous gift from M. Resh (Sloan-Kettering Institute). The plasmids pLKO.1 (Cat. #RHS4078), pLKO.1/eGFP_shRNA_ (Cat. #RHS4459), pLKO.1/TRIM22_shRNA#1_ (Cat. #RHS3979-9574742; GenBank Accession NM_006074, sequence: 5′ cgg agc act cat cta caa gtt ctc gag aac ttg tag atg agt gct ccg 3′) and pLKO.1/TRIM22_shRNA#2_ (Cat. #RHS3979-9574744; GenBank Accession NM_006074, sequence: 5′ gtc acc aaa cat tcc gca taa ctc gag tta tgc gga atg ttt ggt gac 3′) were obtained from Open Biosystems. pLKO.1/scrambled_shRNA_ (Addgene plasmid 1864; sequence 5′ cctaa ggtta agtcg ccctc gctct agcga gggcg actta acctt agg 3′) was obtained from [72] through Addgene. pEIAV (“pEV53B”) was obtained from [73]. pMLV (“pCgp”) was obtained from [74]. The plasmid encoding codon-optimized Gag (p96ZM651gag-opt) (from Drs. Yingying Li, Feng Gao and Beatrice H. Hahn [75]) and the HIV-1 p24 monoclonal antibody (183-H12-5C) (from Dr. Bruce Chesebro and Kathy Wehrly [76],[77],[78]) were obtained through the NIH AIDS Research and Reference Reagent Program, Division of AIDS, NIAID, NIH. Poly/MonoUbiquitin (FK2, #PW8810) and PolyUbiquitin (FK1, #PW8805) antibodies were obtained from Biomol International. Unless otherwise stated, all plasmid transfections were performed using Lipofectamine 2000 (Invitrogen). Co-transfections were performed at a 1∶10 ratio (pR9 or pGag or pGagGFP: pcDNA or pTRIM22 or pT22-C15A/C18A respectively).

### Microarray analysis

RNA was harvested from HOS-CD4/CXCR4 cells in log-phase growth 24 hours after human interferon beta (PBL Biomedical Laboratories Cat. #11420-1) treatment using a Qiagen RNeasy Kit. Labeling of RNA was done using standard techniques as described by Affymetrix (Santa Clara, CA). Two independent RNA samples were hybridized with Affymetrix HG-U133A chips in duplicate. Significance Analysis of Microarray software using a cut-off difference of 2-fold and a false discovery rate of 7% was used to analyze changes in gene activity after human IFNβ treatment via the permuted *t* test method [79]. Microarray data is available upon request.

### Virus production and infections

HIV-1/VSV-G vector particles were generated by calcium phosphate-mediated transfection of 293T cells with three plasmids: p156RRLsinPPTCMVGFPWPRE (encoding the HIV-1 vector segment) [80], pCMVdeltaR9 (the packaging construct), and pMD.G (encoding the VSV-G envelope) [81]. Replication-competent virus (R9 and LAI clones) was generated by calcium phosphate-mediated transfection of 293T cells with pR9 or pLAI plasmids. Virus was quantitated for reverse transcriptase activity using standard protocols [82].

Prior to infection, virus aliquots were digested with DNaseI (0.2 units/µl) for 1 hour at 37°C. Cells were infected with varying amounts of virus for 3 hours at 37°C in a minimal volume of media, after which the media containing virus was replaced with fresh media and the cells were then incubated for the desired time.

### Quantitation of virus and Gag particle release

Virus released into the supernatant was pelleted and lysed as described in [82]. Briefly, 1ml viral supernatants were clarified by centrifugation at 350 g for 10 mins at 4°C and passed through a 0.45 µm filter. Virus was precipitated overnight on wet ice at 4°C using 0.5× volumes of PEG solution (30% PEG 8000, 0.4 M NaCl). Precipitated virus was pelleted by centrifugation at 800 g for 45 minutes at 4°C. Gag particles were prepared as previously described [42],[44]. Briefly, 800 µl of supernatant containing Gag particles were pelleted by centrifugation at 20,000 g for 90 mins at 4°C over a 500 µl cushion of 20% sucrose.

PEG-precipitated virus and sucrose-purified Gag particle pellets were lysed with 75 µl and 30 µl of lysis buffer respectively and 2 µl–5 µl of the lysed pellets was used for Western blotting. Lysis buffer consisted of a fresh mix of Buffer A and Buffer B (2∶1 ratio respectively). Buffer A contained 12.5 ml of 1 M Tris (pH 7.8), 1.25 ml of 0.1 M EDTA (pH 8.0), 1.25 ml of 10% Triton X-100, 250 ml of Glycerol, 0.77 g of DTT and 3.72 g of KCl with the volume brought up to 500 ml with ddH_2_O. Buffer B contained 45 ml of 10% Triton X-100 and 1.63 g KCl with the volume brought up to 500 ml with ddH_2_O.

For analysis of cell lysates, cells were centrifuged at 350 g for 5 mins, washed twice with phosphate-buffered saline (PBS) and lysed with 50 µl of RIPA buffer (50 mM Tris-HCl (pH 7.4), 150 mM NaCl, 1 mM EDTA, 1× Complete Protease Inhibitor (Roche), 1% Triton X-100, 0.1% SDS). Two micrograms of the lysed cell pellets were used for Western blotting.

### Immunoprecipitation and Western blotting

Cells were lysed with cold non-denaturing lysis buffer for 20 mins (20 mM Tris-HCl pH 7.4, 150 mM NaCl, 10 mM EDTA, 0.5% Igepal CA-630 (Sigma), 1× Complete EDTA-free proteinase inhibitor (Roche)) and pre-cleared by centrifugation at 16,000×g for 15 mins. Two micrograms of primary antibody was diluted in 500 µl of cold PBS with 0.01% Triton X-100 and mixed with 15 µl of a 50% protein A-sepharose at 4°C for 1 hour with gentle rocking. Beads were washed 3 times with PBS and once with lysis buffer. 5 µl of 10% BSA was added to the beads and mixed with 600 µg–1 mg of pre-cleared cell lysate in a 500 µl–1 ml volume at 4°C for 2 hours with gentle rocking. Beads were washed 3 times with cold lysis buffer and once with PBS.

Samples for Western blotting were mixed with loading buffer and separated on a 10% SDS-PAGE gel. Protein was transferred to PVDF membrane by wet or semi-dry transfer. Western blotting was carried out by blocking the membrane for 1 hour in 5% skim milk followed by a ∼16 hour incubation with 1∶1000 dilution of primary antibody. Detection was carried out using HRP-labeled secondary antibody (1∶5000 for 30 mins) and the Amersham ECL Plus Western Blotting Detection System. Membranes were exposed to Super RX film (Fujifilm) or scanned using a Storm 860 Phosphorimager (Molecular Dynamics). Densitometric analysis in the linear range of the scanned image was performed using IQMac (v1.2) software. Western blots requiring quantitation were verified using an IRDye-labeled secondary antibody (1∶20,000 for 30 mins) and the Odyssey Infrared Imaging System (LI-COR Biosciences) according to manufacturer's directions.

### Knockdown of TRIM22

Nearly confluent HOS-CD4/CXCR4 cells in 12-well plates (1 ml volume) were transfected with 1.6 µg of pLKO.1, pLKO.1/eGFP_shRNA_ or pLKO.1/TRIM22_shRNA_. Cells were cultured for 16 hours, after which they were washed and treated with increasing concentrations of human IFNβ (PBL Biomedical Laboratories Cat. #11420-1) for 20 hours. The cells were then washed and transfected with 0.16 µg of pR9 (encoding full-length HIV-1). After 16 hours, virus released into the supernatant (800 µl) or contained within 2 µg of the cell lysate were purified and analyzed by Western blot as described above.

Decreased expression of TRIM22 mRNA was verified by Northern blot. Briefly, total RNA was isolated using the RNeasy RNA isolation kit (Qiagen). Twenty micrograms of RNA was separated on a 1.2% formaldehyde agarose gel and transferred to a nitrocellulose membrane. A region in the 3′UTR of TRIM22 mRNA from nucleotides 1523 to 1983 (GenBank Accession #BC035582) was PCR amplified from pCMV-SPORT6/TRIM22 (Open Biosystems Cat. # MHS1010-7508483) and random-labeled with αP^32^ for use as a probe. Differences in loading amounts were assessed using a β-actin mRNA probe end-labeled with γP^32^. Membranes were analyzed using a Storm 860 Phosphorimager.

### Gag localization

HOS-CD4/CXCR4 cells cultured in 12-well plates on 18mm coverslips were co-transfected with pTRIM22 and pGag/GFP (10∶1 ratio respectively) for 3 hours, washed and then treated with 100 µg/ml of cycloheximide (Sigma) for 3 hours as described in [44]. Cells were incubated in fresh media to release the cycloheximide block and incubated for 48 hours. The coverslips containing the cells were then washed twice with PBS, fixed for 10 mins in PBS containing 5% formaldehyde and 2% sucrose and then washed twice more with PBS. Coverslips were mounted onto glass slides with ∼15 µl of Vectashield (Vector Laboratories) containing 0.5 mg/ml DAPI stain and then sealed with nail polish. Slides were examined using 40× oil immersion using a Zeiss fluorescence microscope fitted with an Apotome.

### Myristoylation assay

HOS cells ∼90% confluent in 6-well plates were co-transfected with 0.4 µg pGag and 4 µg of pcDNA3.1 or pTRIM22 for 24 hours. Cells were then labeled with 100 µCi/ml of ^3^H-myristic acid (Perkin Elmer) for 16 hours. Cells were washed 4 times with PBS and subjected to immunoprecipitation using p24CA antibodies as described above. Samples were resolved on a 10% SDS-PAGE gel, impregnated with EN^3^HANCE scintillant (Perkin Elmer), dried and exposed to a phosphorimager screen for 3 days at room temperature. The screen was analyzed using a Storm 860 Phosphorimager and IQMac (v1.2) software.

### Pulse-chase assay

The pulse-chase stability assay for Gag was carried out as previously described with some modifications [13]. Nearly confluent 10 cm dishes containing HOS-CD4/CXCR4 cells were transfected with 30 µg of pcDNA3.1 or pTRIM22 and 3 µg of pGag-opt. After 36 hours, cells were washed with PBS and then incubated in methionine-, cystine- and cysteine-free DMEM (MP Biomedicals) for 1 hour. Cells were labeled for 45 mins with 50 µCi/ml of [^35^S] EasyTag Express Protein Labeling Mix (Perkin Elmer), washed and incubated in standard DMEM growth media with 10% heat-inactivated FBS for 0, 1, 2 or 4 hours. Cells were processed for immunoprecipitation with monoclonal p24 antibodies as described earlier. Precipitated proteins were separated on a 10% SDS-PAGE gel, impregnated with EN3HANCE scintillant (Perkin Elmer), dried and analyzed by phosphorimager.

## Supporting Information

Table S1 Top 52 probe sets affected by interferon beta treatment of HOS-CD4/CXCR4 cells.
(0.02 MB XLS)

## References

[1] Sen GC (2001). Viruses and interferons.. Annu Rev Microbiol.

[2] Karpov AV (2001). Endogenous and exogenous interferons in HIV-infection.. Eur J Med Res.

[3] Stark GR, Kerr IM, Williams BRG, Silverman RH, Schreiber RD (1998). How Cells Respond to Interferons.. Annu Rev Biochem.

[4] Der SD, Zhou A, Williams BR, Silverman RH (1998). Identification of genes differentially regulated by interferon alpha, beta, or gamma using oligonucleotide arrays.. Proc Natl Acad Sci USA.

[5] Agy MB, Acker RL, Sherbert CH, Katze MG (1995). Interferon treatment inhibits virus replication in HIV-1 and SIV-infected CD4+ T-cell lines by distinct mechanisms: evidence for decreased stability and aberrant processing of HIV-1 proteins.. Virology.

[6] Coccia EM, Krust B, Hovanessian AG (1994). Specific inhibition of viral protein synthesis in HIV-infected cells in response to interferon treatment.. J Biol Chem.

[7] Baca-Regen L, Heinzinger N, Stevenson M, Gendelman HE (1994). Alpha interferon-induced antiretroviral activities: restriction of viral nucleic acid synthesis and progeny virion production in human immunodeficiency virus type 1-infected monocytes.. J Virol.

[8] Hansen BD, Nara PL, Maheshwari RK, Sidhu GS, Bernbaum JG (1992). Loss of infectivity by progeny virus from alpha interferon-treated human immunodeficiency virus type 1-infected T cells is associated with defective assembly of envelope gp120.. J Virol.

[9] Fernie BF, Poli G, Fauci AS (1991). Alpha interferon suppresses virion but not soluble human immunodeficiency virus antigen production in chronically infected T-lymphocytic cells.. J Virol.

[10] Shirazi Y, Pitha PM (1992). Alpha interferon inhibits early stages of the human immunodeficiency virus type 1 replication cycle.. J Virol.

[11] Okumura A, Lu G, Pitha-Rowe I, Pitha PM (2006). Innate antiviral response targets HIV-1 release by the induction of ubiquitin-like protein ISG15.. Proc Natl Acad Sci U S A.

[12] Sokolskaja E, Luban J (2006). Cyclophilin, TRIM5, and innate immunity to HIV-1.. Curr Opin Microbiol.

[13] Sakuma R, Noser JA, Ohmine S, Ikeda Y (2007). Rhesus monkey TRIM5alpha restricts HIV-1 production through rapid degradation of viral Gag polyproteins.. Nat Med.

[14] Nisole S, Stoye JP, Saib A (2005). TRIM family proteins: retroviral restriction and antiviral defence.. Nat Rev Microbiol.

[15] Wolf D, Goff SP (2007). TRIM28 Mediates Primer Binding Site-Targeted Silencing of Murine Leukemia Virus in Embryonic Cells.. Cell.

[16] Saurin AJ, Borden KL, Boddy MN, Freemont PS (1996). Does this have a familiar RING?. Trends Biochem Sci.

[17] Xu L, Yang L, Moitra PK, Hashimoto K, Rallabhandi P (2003). BTBD1 and BTBD2 colocalize to cytoplasmic bodies with the RBCC/tripartite motif protein, TRIM5delta.. Exp Cell Res.

[18] Trockenbacher A, Suckow V, Foerster J, Winter J, Krauss S (2001). MID1, mutated in Opitz syndrome, encodes an ubiquitin ligase that targets phosphatase 2A for degradation.. Nat Genet.

[19] Urano T, Saito T, Tsukui T, Fujita M, Hosoi T (2002). Efp targets 14-3-3 sigma for proteolysis and promotes breast tumour growth.. Nature.

[20] Gack MU, Shin YC, Joo CH, Urano T, Liang C (2007). TRIM25 RING-finger E3 ubiquitin ligase is essential for RIG-I-mediated antiviral activity.. Nature.

[21] Horn EJ, Albor A, Liu Y, El-Hizawi S, Vanderbeek GE (2004). RING protein Trim32 associated with skin carcinogenesis has anti-apoptotic and E3-ubiquitin ligase properties.. Carcinogenesis.

[22] Vichi A, Payne DM, Pacheco-Rodriguez G, Moss J, Vaughan M (2005). E3 ubiquitin ligase activity of the trifunctional ARD1 (ADP-ribosylation factor domain protein 1).. Proc Natl Acad Sci U S A.

[23] Reymond A, Meroni G, Fantozzi A, Merla G, Cairo S (2001). The tripartite motif family identifies cell compartments.. Embo J.

[24] Hilton DJ, Richardson RT, Alexander WS, Viney EM, Willson TA (1998). Twenty proteins containing a C-terminal SOCS box form five structural classes.. Proc Natl Acad Sci U S A.

[25] Ponting C, Schultz J, Bork P (1997). SPRY domains in ryanodine receptors (Ca(2+)-release channels).. Trends Biochem Sci.

[26] Tissot C, Mechti N (1995). Molecular cloning of a new interferon-induced factor that represses human immunodeficiency virus type 1 long terminal repeat expression.. J Biol Chem.

[27] Bouazzaoui A, Kreutz M, Eisert V, Dinauer N, Heinzelmann A (2006). Stimulated trans-acting factor of 50 kDa (Staf50) inhibits HIV-1 replication in human monocyte-derived macrophages.. Virology.

[28] Bandman O, Coleman RT, Loring JF, Seilhamer JJ, Cocks BG (2002). Complexity of inflammatory responses in endothelial cells and vascular smooth muscle cells determined by microarray analysis.. Ann N Y Acad Sci.

[29] Obad S, Olofsson T, Mechti N, Gullberg U, Drott K (2007). Expression of the IFN-inducible p53-target gene TRIM22 is down-regulated during erythroid differentiation of human bone marrow.. Leuk Res.

[30] Deng YJ, Huang ZX, Zhou CJ, Wang JW, You Y (2006). Gene profiling involved in immature CD4+ T lymphocyte responsible for systemic lupus erythematosus.. Mol Immunol.

[31] Zirn B, Hartmann O, Samans B, Krause M, Wittmann S (2006). Expression profiling of Wilms tumors reveals new candidate genes for different clinical parameters.. Int J Cancer.

[32] Gongora C, Tissot C, Cerdan C, Mechti N (2000). The interferon-inducible Staf50 gene is downregulated during T cell costimulation by CD2 and CD28.. J Interferon Cytokine Res.

[33] Zhang J, Das SC, Kotalik C, Pattnaik AK, Zhang L (2004). The latent membrane protein 1 of Epstein-Barr virus establishes an antiviral state via induction of interferon-stimulated genes.. J Biol Chem.

[34] Renne R, Barry C, Dittmer D, Compitello N, Brown PO (2001). Modulation of cellular and viral gene expression by the latency-associated nuclear antigen of Kaposi's sarcoma-associated herpesvirus.. J Virol.

[35] Izmailova E, Bertley FM, Huang Q, Makori N, Miller CJ (2003). HIV-1 Tat reprograms immature dendritic cells to express chemoattractants for activated T cells and macrophages.. Nat Med.

[36] Asaoka K, Ikeda K, Hishinuma T, Horie-Inoue K, Takeda S (2005). A retrovirus restriction factor TRIM5alpha is transcriptionally regulated by interferons.. Biochem Biophys Res Commun.

[37] Chelbi-Alix MK, Pelicano L, Quignon F, Koken MH, Venturini L (1995). Induction of the PML protein by interferons in normal and APL cells.. Leukemia.

[38] Orimo A, Tominaga N, Yoshimura K, Yamauchi Y, Nomura M (2000). Molecular cloning of ring finger protein 21 (RNF21)/interferon-responsive finger protein (ifp1), which possesses two RING-B box-coiled coil domains in tandem.. Genomics.

[39] Rhodes DA, Ihrke G, Reinicke AT, Malcherek G, Towey M (2002). The 52 000 MW Ro/SS-A autoantigen in Sjogren's syndrome/systemic lupus erythematosus (Ro52) is an interferon-gamma inducible tripartite motif protein associated with membrane proximal structures.. Immunology.

[40] Garoff H, Hewson R, Opstelten DJ (1998). Virus maturation by budding.. Microbiol Mol Biol Rev.

[41] Hermida-Matsumoto L, Resh MD (2000). Localization of human immunodeficiency virus type 1 Gag and Env at the plasma membrane by confocal imaging.. J Virol.

[42] Pornillos O, Higginson DS, Stray KM, Fisher RD, Garrus JE (2003). HIV Gag mimics the Tsg101-recruiting activity of the human Hrs protein.. J Cell Biol.

[43] Garrus JE, von Schwedler UK, Pornillos OW, Morham SG, Zavitz KH (2001). Tsg101 and the vacuolar protein sorting pathway are essential for hiv-1 budding.. Cell.

[44] Perlman M, Resh MD (2006). Identification of an intracellular trafficking and assembly pathway for HIV-1 gag.. Traffic.

[45] Furuishi K, Matsuoka H, Takama M, Takahashi I, Misumi S (1997). Blockage of N-myristoylation of HIV-1 gag induces the production of impotent progeny virus.. Biochem Biophys Res Commun.

[46] Morikawa Y, Hinata S, Tomoda H, Goto T, Nakai M (1996). Complete inhibition of human immunodeficiency virus Gag myristoylation is necessary for inhibition of particle budding.. J Biol Chem.

[47] Pal R, Reitz MS, Tschachler E, Gallo RC, Sarngadharan MG (1990). Myristoylation of gag proteins of HIV-1 plays an important role in virus assembly.. AIDS Res Hum Retroviruses.

[48] Bryant M, Ratner L (1990). Myristoylation-dependent replication and assembly of human immunodeficiency virus 1.. Proc Natl Acad Sci U S A.

[49] Tashiro A, Shoji S, Kubota Y (1989). Antimyristoylation of the gag proteins in the human immunodeficiency virus-infected cells with N-myristoyl glycinal diethylacetal resulted in inhibition of virus production.. Biochem Biophys Res Commun.

[50] Hicke L (2001). Protein regulation by monoubiquitin.. Nat Rev Mol Cell Biol.

[51] Kerscher O, Felberbaum R, Hochstrasser M (2006). Modification of proteins by ubiquitin and ubiquitin-like proteins.. Annu Rev Cell Dev Biol.

[52] Waterman H, Levkowitz G, Alroy I, Yarden Y (1999). The RING finger of c-Cbl mediates desensitization of the epidermal growth factor receptor.. J Biol Chem.

[53] Stremlau M, Owens CM, Perron MJ, Kiessling M, Autissier P (2004). The cytoplasmic body component TRIM5alpha restricts HIV-1 infection in Old World monkeys.. Nature.

[54] Finzi A, Orthwein A, Mercier J, Cohen EA (2007). Productive human immunodeficiency virus type 1 assembly takes place at the plasma membrane.. J Virol.

[55] Welsch S, Keppler OT, Habermann A, Allespach I, Krijnse-Locker J (2007). HIV-1 buds predominantly at the plasma membrane of primary human macrophages.. PLoS Pathog.

[56] Poli G, Orenstein JM, Kinter A, Folks TM, Fauci AS (1989). Interferon-alpha but not AZT suppresses HIV expression in chronically infected cell lines.. Science.

[57] Yasuda Y, Miyake S, Kato S, Kita M, Kishida T (1990). Interferon-alpha treatment leads to accumulation of virus particles on the surface of cells persistently infected with the human immunodeficiency virus type 1.. J Acquir Immune Defic Syndr.

[58] Crespi M (1989). The effect of interferon on cells persistently infected with HIV.. Aids.

[59] Smith MS, Thresher RJ, Pagano JS (1991). Inhibition of human immunodeficiency virus type 1 morphogenesis in T cells by alpha interferon.. Antimicrob Agents Chemother.

[60] Dianzani F, Castilletti C, Gentile M, Gelderblom HR, Frezza F (1998). Effects of IFN alpha on late stages of HIV-1 replication cycle.. Biochimie.

[61] Gendelman HE, Baca LM, Turpin J, Kalter DC, Hansen B (1990). Regulation of HIV replication in infected monocytes by IFN-alpha. Mechanism for viral restriction.. Journal of Immunology.

[62] Gendelman HE, Baca L, Turpin JA, Kalter DC, Hansen BD (1990). Restriction of HIV replication in infected T cells and monocytes by interferon-alpha.. AIDS Res Hum Retroviruses.

[63] Sakuma R, Noser JA, Ohmine S, Ikeda Y (2007). Rhesus monkey TRIM5alpha restricts HIV-1 production through rapid degradation of viral Gag polyproteins.. Nat Med.

[64] Nakasato N, Ikeda K, Urano T, Horie-Inoue K, Takeda S (2006). A ubiquitin E3 ligase Efp is up-regulated by interferons and conjugated with ISG15.. Biochem Biophys Res Commun.

[65] Wong JJ, Pung YF, Sze NS, Chin KC (2006). HERC5 is an IFN-induced HECT-type E3 protein ligase that mediates type I IFN-induced ISGylation of protein targets.. Proc Natl Acad Sci U S A.

[66] Zou W, Zhang DE (2006). The interferon-inducible ubiquitin-protein isopeptide ligase (E3) EFP also functions as an ISG15 E3 ligase.. J Biol Chem.

[67] Neil SJ, Sandrin V, Sundquist WI, Bieniasz PD (2007). An interferon-alpha-induced tethering mechanism inhibits HIV-1 and Ebola virus particle release but is counteracted by the HIV-1 Vpu protein.. Cell Host Microbe.

[68] Spearman P, Horton R, Ratner L, Kuli-Zade I (1997). Membrane binding of human immunodeficiency virus type 1 matrix protein in vivo supports a conformational myristyl switch mechanism.. J Virol.

[69] Resh MD (2004). A myristoyl switch regulates membrane binding of HIV-1 Gag.. Proc Natl Acad Sci U S A.

[70] Tang C, Loeliger E, Luncsford P, Kinde I, Beckett D (2004). Entropic switch regulates myristate exposure in the HIV-1 matrix protein.. Proc Natl Acad Sci U S A.

[71] Wu Z, Alexandratos J, Ericksen B, Lubkowski J, Gallo RC (2004). Total chemical synthesis of N-myristoylated HIV-1 matrix protein p17: structural and mechanistic implications of p17 myristoylation.. Proc Natl Acad Sci U S A.

[72] Sarbassov DD, Guertin DA, Ali SM, Sabatini DM (2005). Phosphorylation and regulation of Akt/PKB by the rictor-mTOR complex.. Science.

[73] Olsen JC (1998). Gene transfer vectors derived from equine infectious anemia virus.. Gene Ther.

[74] Han JY, Cannon PM, Lai KM, Zhao Y, Eiden MV (1997). Identification of envelope protein residues required for the expanded host range of 10A1 murine leukemia virus.. J Virol.

[75] Gao F, Li Y, Decker JM, Peyerl FW, Bibollet-Ruche F (2003). Codon usage optimization of HIV type 1 subtype C gag, pol, env, and nef genes: in vitro expression and immune responses in DNA-vaccinated mice.. AIDS Res Hum Retroviruses.

[76] Wehrly K, Chesebro B (1997). p24 antigen capture assay for quantification of human immunodeficiency virus using readily available inexpensive reagents.. Methods.

[77] Chesebro B, Wehrly K, Nishio J, Perryman S (1992). Macrophage-tropic human immunodeficiency virus isolates from different patients exhibit unusual V3 envelope sequence homogeneity in comparison with T-cell-tropic isolates: definition of critical amino acids involved in cell tropism.. J Virol.

[78] Toohey K, Wehrly K, Nishio J, Perryman S, Chesebro B (1995). Human immunodeficiency virus envelope V1 and V2 regions influence replication efficiency in macrophages by affecting virus spread.. Virology.

[79] Tusher VG, Tibshirani R, Chu G (2001). Significance analysis of microarrays applied to the ionizing radiation response.. Proc Natl Acad Sci USA.

[80] Follenzi A, Ailes LE, Bakovic S, Gueuna M, Naldini L (2000). Gene transfer by lentiviral vectors is limited by nuclear translocation and rescued by HIV-1 pol sequences.. Nat Genetics.

[81] Naldini L, Blomer U, Gallay P, Ory D, Mulligan R (1996). In Vivo Gene Delivery and Stable Transduction of Nondividing Cells by a Lentiviral Vector.. Science.

[82] Johnson VA, Byington RE, Kaplan JC, Aldovini A, Walker BD (1990). Reverse Transcriptase (RT) Assay.. Techniques in HIV research.

